# Trends in molecular subtypes of breast cancer: description of incidence rates between 2007 and 2012 from three French registries

**DOI:** 10.1186/s12885-018-4080-8

**Published:** 2018-02-07

**Authors:** Marion Cortet, Aurélie Bertaut, Florence Molinié, Simona Bara, Françoise Beltjens, Charles Coutant, Patrick Arveux

**Affiliations:** 1 Département d’oncologie chirurgicale, Georges-François Leclerc Cancer Centre, UNICANCER, 1 rue du Professeur Marion, 21000 Dijon, France; 2 Breast and Gynaecologic Cancer Registry of Côte d’Or, Georges-François Leclerc Cancer Centre, UNICANCER, 1 rue du Professeur Marion, 21000 Dijon, France; 3 Registre des Cancers de Loire-Atlantique, Centre Hospitalier Universitaire de Nantes, 50 route de Saint Sébastien, 44093 Nantes, France; 4 Registre des Cancers de la Manche, Centre Hospitalier du Cotentin, 46 rue du Val de Saire, 50102 Cherbourg, France; 5 Département de pathologie, Georges-François Leclerc Cancer Centre, UNICANCER, 1 rue du Professeur Marion, 21000 Dijon, France; 6 Centre for Research in Epidemiology and Population Health (CESP), “Health across Generations” Team, Gustave Roussy, Inserm U1018, Villejuif, France

**Keywords:** Incidence, Breast cancer, Population data

## Abstract

**Background:**

The incidence and incidence trends of breast cancer according to molecular subtype are unknown at a population level in France. The registry data enables this study and may give this information, that is crucial to describe and understand breast cancer epidemiology.

**Methods:**

We estimated the incidence rates of breast cancer for each molecular subtype using data from three cancer registries in France for the period from 2007 to 2012. Molecular subtypes were defined with immunohistochemical data. Poisson models were estimated to modelize the course of breast cancer incidence and to test the trends.

**Results:**

The study included 12,040 patients diagnosed between 2007 and 2012 in the three administrative areas covered by the registries. There was no significant trends in the proportion of each molecular subtype year by year. The age distribution of incident cases was different depending on the molecular subtypes (*p* < 0.001). The course of incidence between 2007 and 2012 was also different depending on molecular subtype according to the multivariate Poisson model (*p* < 0.001).

**Conclusion:**

The description of incident cases of breast cancer according to molecular subtype at a population level showed differences in trends. The trends in incidence differed according to molecular subtype, and this should improve our understanding of overall changes in incidence. This analysis is important to plan screening and treatment resources at a population level.

## Background

Breast cancer is the most frequent cancer in women in France [1]. Trends in incidence are well described [1], although explanations for these trends are lacking. Epidemiological studies have failed to propose explanations for observed trends in breast cancer incidence. However, help may be at hand from biological researchers. Indeed, breast cancer is now defined according to molecular subtypes [2], which can be roughly identified by routine immunohistochemical exams, namely hormonal receptors (HR) and Human epidermal growth factor receptor (HER2) [3]. The molecular subtypes identified correspond to different disease profiles within the overall heading of breast cancer. Their epidemiological features are not the same, with different risk factors, recurrence patterns, recurrence rates and survival curves. Additionally, management is not the same, and treatment now depends on the molecular subtype.

There is still few population-based epidemiological studies on this subject, since immunohistochemical characteristics are rarely available at a population level. HER2 status is available in the Surveillance, Epidemiology and End Result program (SEER) registries since 2010, and HR status since slightly earlier. Descriptions of incidence according to molecular subtypes were reported by the SEER [4, 5] for two years (2010, 2011), corresponding to the first years with available HER2 status. One study was conducted in Norway, with estimation of secular trends, with imputation for unknown molecular subtypes [6]. However, to the best of our knowledge, no study exists to date reporting trends in the incidence of breast cancer according to molecular subtype from a population perspective.

French cancer registries systematically record precise information about each case. HR and HER2 status have been recorded since 2007 in three French cancer registries, namely th registries of the Manche, Loire-Atlantique and Côte d’Or Departments of France.

Therefore, this study aimed to describe the trends in breast cancer incidence according to molecular subtype at a population level.

## Materials and methods

### Population

Data from three French cancer registries were used (Manche, Loire Atlantique and Côte d’Or). These registries perform exhaustive registration of all new cases of breast cancer diagnosed within their administrative area. The population of these three administrative areas gather 2,300,000 inhabitants. These three population-based registries are labelled by the INSERM (Institut National de la Santé et de la Recherche Médicale), the Santé Publique France Agency, and the National Cancer Institute (INCA). The mortality/incidence ratio is 20.7 and the microscopic verification proportion is 99.5% for female invasive breast cancer in the Loire-Atlantique cancer registry (2012–14).

The mortality/incidence ratio is 19.4 and the microscopic verification proportion is 98.2% for female invasive breast cancer in the Manche cancer registry (2012–14).

All patients diagnosed with an invasive breast cancer between 2007 and 2012 were included in the study (*n* = 12,040 patients).

### Data

Year of diagnosis and age at diagnosis were available in the registry data. Age classes were computed in four groups for the population description, and then by five-year intervals for incidence standardization. Immunohistochemical data such as oestrogen receptor (ER), progesterone receptor (PR) and human epidermal growth factor receptor (HER2) were recorded for each new breast cancer case. If HER2 expression status was uncertain by immunohistochemistry, analysis by fluorescence in situ hybridization (FISH) was performed. Immunohistochemical analyses and FISH analyses were performed in routine laboratories, no special analysis was performed for this study. Molecular subtypes were defined as follows: triple negative (TN) (HER2 negative, ER negative and PR negative), HR+/HER2- (HER2 negative, ER positive, PR positive or negative), HR+/HER2+ (HER2 positive, ER positive, PR positive or negative), HR-/HER2+ (HER2 positive, ER negative, PR negative). For 1683 patients, molecular subtype was not defined because of missing data on immunohistochemical analyses. They were included as NA (not available) in the univariate description, but they were excluded from the Poisson model.

The population composition by age and gender for each administrative area and for each year was obtained from the National Institute of Statistics and Economic Studies (Institut National des Statistiques et des Etudes Economiques, INSEE).

### Statistical analysis

Descriptive analysis of available data was performed. Crude incidence rates were estimated year by year, for the overall cases, and then by molecular subtype. Standardized incidence rates were estimated, according to the World standard population from the World Health Organization (WHO) [7] . Standardized incidence rates were also estimated by year, for the overall population and for each molecular subtype.

Poisson models were used to analyse the incidence rates by year, age class and molecular subtype. Interaction was tested by the likelihood ratio test. Bsplines were used to adjust semi-quantitative variable such as age class.

Statistical analyses were performed with R 3.2.2 software [8].

## Results

Overall, 12,040 incident cases of breast cancer were included in the three French departments between 2007 and 2012 (Table 1). Among all cancer cases, 4.5% were diagnosed before the age of 40, and 30.5% after 70 years of age.

**Table 1:** Description of the population of patients with breast cancer diagnosed between 2007 and 2012 in three French administrative areas

Variable	N = 12,040	%
Year		
2007	1976	16.4
2008	1940	16.1
2009	1910	15.9
2010	2069	17.2
2011	2063	17.1
2012	2082	17.3
Age class		
< 40	547	4.5
40–54	3447	28.6
55–69	4377	36.4
> = 70	3669	30.5
Biomarker		
ER+ (699 NA, 6%)	9492	78.8
PR+ (707 NA, 6%)	8144	67.6
HER2+ (1524 NA, 13%)	1239	10.3
Molecular subtype		
TN	1111	9.2
HR+/HER2+	758	6.3
HR+/HER2-	8041	66.8
HR-/HER2+	447	3.7
NA	1683	14.0

ER: estrogen receptor, PR: progesterone receptor, HER2: human epidermal growth factor receptor, TN: triple negative; HR: hormonal receptor; NA: not available

The proportion of ER positive tumours was high (78.8%). All PR-positive tumours were ER positive; 10.3% of tumours were positive for HER2.

Estimated incidence rates are presented in Table 2. HR+/ HER2-cancers were the most frequent. The lowest standardized incidence rate (SIR) was in 2009, and the highest in 2010 for the overall population.

**Table 2:** Crude and world- and Europe standardized incidence rates (per 10^5^ person-years) for the overall population and for each molecular subtype, by year, from 2007 to 2012

Year of diagnosis	2007	2008	2009	2010	2011	2012
Crude incidence rate (IR)						
Overall	170.99	166.88	159.45	177.99	170.83	171.37
TN	15.88	16.80	14.23	13.70	16.28	16.48
HR+/HER2+	9.59	11.10	10.36	11.41	9.38	13.00
HR+/HER2-	110.23	106.95	107.13	121.43	118.90	114.76
HR-/HER2+	5.65	5.28	5.65	6.75	6.59	6.45
World Standardized IR						
Overall	116.84	113.77	109.67	119.35	114.59	113.89
TN	11.96	13.00	10.43	9.85	11.22	12.00
HR+/HER2+	6.70	8.26	8.37	8.74	7.26	10.03
HR+/HER2-	75.78	73.03	72.73	82.07	79.68	75.21
HR-/HER2+	4.10	4.08	4.53	5.00	4.94	4.48
Europe Standardized IR						
Overall	154.27	149.78	142.02	157.86	150.87	149.10
TN	15.35	15.95	13.11	12.52	13.85	14.85
HR+/HER2+	8.37	10.65	10.20	11.18	9.32	11.88
HR+/HER2-	100.97	96.98	95.48	108.85	106.15	100.69
HR-/HER2+	4.90	4.99	5.35	6.33	6.09	5.61

TN: Triple Negative; HR: Hormone Receptors; HER: Human epidermal growth factor

**Fig. 1:**
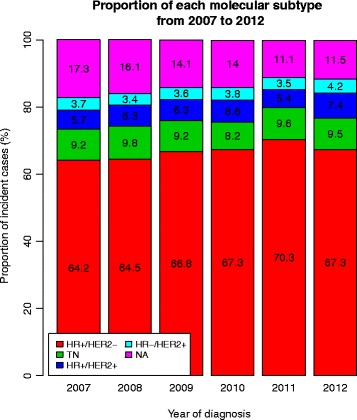
Proportion of each molecular subtype among breast cancer cases diagnosed between 2007 and 2012

**Fig. 2:**
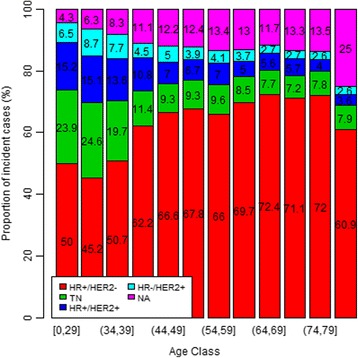
Proportion of each molecular subtype by age class among breast cancer cases diagnosed between 2007 and 2012

Figure 1 presents the proportion of each molecular subtype among cancers for each year. Figure 2 presents the proportion of each molecular subtype, by age class. The frequency of cancers with unknown subtype decreased with year of diagnosis. There were more unknown types among elderly patients. The proportion of triple negative cancers was higher in younger patients than in elderly patients, while the proportion of HER2+ tumours decreased with age.

**Fig. 3:**
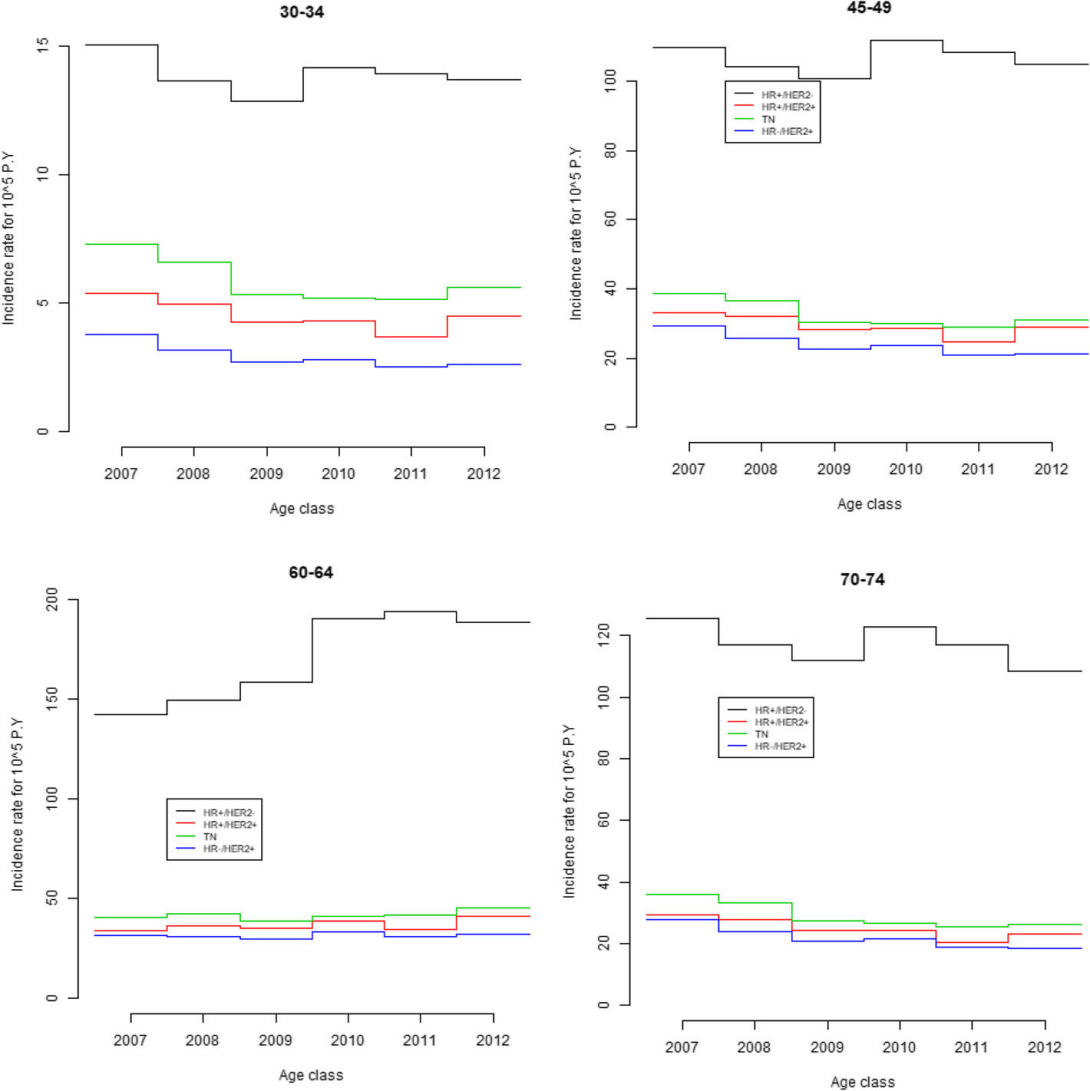
Incidence rates of breast cancer according to molecular subtype from 2007 to 2012, by age class, estimated by Poisson regression.The Model was adjusted on age class, year of diagnosis, molecular subtype, interaction between molecular subtype and age class and between molecular subtype and year of diagnosis. Black: HR+/HER2-, red: HR+/HER2+, green: Triple negative, BlueHR-/HER2+

Using Poisson models, we analysed the incidence rates by age class, molecular subtype and year of diagnosis. The final model is adjusted for year of diagnosis, molecular subtype and patient age. It also includes a term for the interaction of the year of diagnosis with molecular subtype (*p* < 0.001), as well as a term for the interaction of age class with molecular subtype (*p* < 0.001), since these interactions were significant using likelihood ratio tests. The trend in incidence was therefore different among age classes according to molecular subtype. The course of incidence from 2007 to 2012 also significantly differed depending on the molecular subtype according to this model. The incidence rates obtained by Poisson modelisation by by year, according to the different molecular subtypes and for each age class are described in Fig. 3. Only four age classes are described. The other age class are available in Appendix 1. HR+/HER2- tumours incidence does not seem to change except for 60–64 age class, where it seems to increase with time. The other molecular subtypes tend to decrease in incidence, except for the 60–64 age class. This decreasement is not confirmed in 2012 diagnosis year.

## Discussion

Our study is based on population data from established and exhaustive cancer registries. For this study, molecular information about breast tumours was recorded, and these data about molecular subtype are now recognized as useful for predicting prognosis and therefore, for making treatment decisions. The results show different evolution of molecular profile incidence among age class and among the studied years of diagnosis.

The strength of this study is the report of valid population data, with a good registration of incident cases. Nevertheless, the immunohistochemical data were not available for all incident cases. This missing data were more frequent in early years and for old patients. The SEER reports [4, 5] had missing data about immunohistochemical analyses for 12% of patients in 2010. The report from Norway [6] produced imputed estimation. Here, missing data were described by age class and by year of diagnosis, to show that they were not missing at random. Missing data were more frequent in first year of immunohistochemical data registration and for elderly patients.

The spread of the different molecular subtypes is similar to that described by Howlader and Kohler [4, 5] in the SEER study. In their article, Howlader et al. found 73% Luminal A tumours, defined as HR+/HER2- tumours (versus 77.6% in our study), 12% triple-negative tumours (versus 10.7% in our study), 5% HER 2 positive tumours with negative hormone receptors (versus 4.3%) and 10% HR+/HER2 + tumours (versus 7.3% in our study). These two studies, both conducted on population databases, thus reported coherent results, and both reported a different spread in molecular subtypes than that described in clinical trials, where HER2 positive tumours are described as being much more frequent than in population databases (15–30% vs 11.8%) [9]. Indeed, HER2 positive tumours are probably more exposed to inclusion in clinical trial since they are probably more frequently managed in cancer comprehensive cancer.

The profile of incidence by age class and by molecular subtype also differed compared to that described in the United States, where the age class with the maximal incidence is around 65–75 [4], compared to age 50–60 in France. This may be due to nationwide implementation of mass screening for breast cancer from age 50 onwards in France. For the administrative areas described here, the participation rate to the national screening programme is between 52% and 55%. This national program is for women aged between 50 and 75 years-old.

The course of the trends in breast cancer incidence may be at least partially explained by the incidence of each molecular subtype. Indeed, several studies have shown that the distribution of molecular subtypes depends on ethnicity as well as environmental risk factors [10] even if a recent French study found no significant association between socioeconomic status and molecular subtype [11] . Ethnicity and environmental risk factors were not studied here but the differences in incidence trends between the molecular subtypes may be explained by changes in environmental factors.

Treatment of breast cancer is now adapted according to the molecular subtype, and recurrence-free survival differs among the different subtypes, both in terms of time to recurrence, and type of recurrence [12]. The analysis of incidence and prognosis at a population level should take into account these molecular subtypes to evaluate and compare patient survival and to plan ressources needed for treatment.

These different molecular subtypes have different risk factors, and a different distribution across age classes. Some studies showed that the proportion of luminal A subtype increased with age class [13], whereas triple negative tumours decreased with age. In absolute incidence rates, these findings were not observed in our study.

This study aimed to describe the trends in incidence and distribution among age classes of breast cancers by molecular subtypes. The models used show an increasing proportion of luminal non HER2 cancers between 2007 and 2012, the one targeted by the screening program since they progress slowly. The effect of age class was also significantly different depending on the molecular subtype in our study. First, the overall trends show a decrease in breast cancer incidence in 2009. This decrease corresponds to a decreasing proportion of cancers diagnosed in the screening program.

## Conclusions

The overall trends in incidence of breast cancer may be explained by the trends in incidence of molecular subtypes. This study shows significant differences in the age distribution by molecular subtype, and in year-on-year trends between molecular subtypes. Further studies are needed to understand the differences observed here. This analysis is crucial for the planning of screening, diagnosis and treatment resources for breast cancer at a population level.

## References

[1] Binder-Foucard F, Bossard N, Delafosse P, Belot A, Woronoff A-S, Remontet L (2014). Cancer incidence and mortality in France over the 1980-2012 period: solid tumors. Rev Dépidémiologie Santé Publique.

[2] Perou CM, Sørlie T, Eisen MB, van de Rijn M, Jeffrey SS, Rees CA (2000). Molecular portraits of human breast tumours. Nature.

[3] Prat A, Carey LA, Adamo B, Vidal M, Tabernero J, Cortés J, et al. Molecular features and survival outcomes of the intrinsic subtypes within HER2-positive breast cancer. J Natl Cancer Inst. 2014;106(8). 10.1093/jnci/dju152. 10.1093/jnci/dju152 PMC4151853 25139534

[4] Howlader N, Altekruse SF, Li CI, Chen VW, Clarke CA, Ries LAG, et al. US incidence of breast cancer subtypes defined by joint hormone receptor and HER2 status. J Natl Cancer Inst. 2014;106(5). 10.1093/jnci/dju055. 10.1093/jnci/dju055 PMC4580552 24777111

[5] Kohler BA, Sherman RL, Howlader N, Jemal A, Ryerson AB, Henry KA (2015). Annual Report to the Nation on the Status of Cancer, 1975–2011, Featuring Incidence of Breast Cancer Subtypes by Race/Ethnicity, Poverty, and State. J. Natl. Cancer Inst.

[6] Valla M, Vatten LJ, Engstrøm MJ, Haugen OA, Akslen LA, Bjørngaard JH (2016). Molecular subtypes of breast cancer: long-term incidence trends and prognostic differences. Cancer Epidemiol Biomark Prev Publ Am Assoc Cancer Res Cosponsored Am Soc Prev Oncol.

[7] Ahmad OB, Boschi-Pinto C, Lopez AD, Murray CJ, Lozano R, Inoue M (2001). Age standardization of rates: a new WHO standard [Internet].

[8] R Core Team (2014). R: A Language and Environment for Statistical Computing [Internet].

[9] Anderson WF, Rosenberg PS, Katki HA. Tracking and evaluating molecular tumor markers with cancer registry data: HER2 and breast cancer. J Natl Cancer Inst. 2014;106 10.1093/jnci/dju093 PMC4677264 24777110

[10] Devi CRB, Tang TS, Corbex M (2012). Incidence and Risk factors for breast cancer subtypes in three distinct south-east Asian ethnic groups: Chinese, Malay and natives of Sarawak, Malaysia. Int. J. Cancer J. Int. Cancer.

[11] Auguste A, Cortet M, Dabakuyo-Yonli TS, Launay L, Arnould L, Desmoulins I (2017). Breast cancer subtype of French women is not influenced by socioeconomic status: a population-based-study. PLoS One.

[12] Metzger-Filho O, Sun Z, Viale G, Price KN, Crivellari D, Snyder RD (2013). Patterns of recurrence and outcome according to breast cancer subtypes in lymph node-negative disease: results from international breast cancer study group trials VIII and IX. J Clin Oncol Off J Am Soc Clin Oncol.

[13] Jenkins EO, Deal AM, Anders CK, Prat A, Perou CM, Carey LA (2014). Age-specific changes in intrinsic breast cancer subtypes: a focus on older women. Oncologist.

